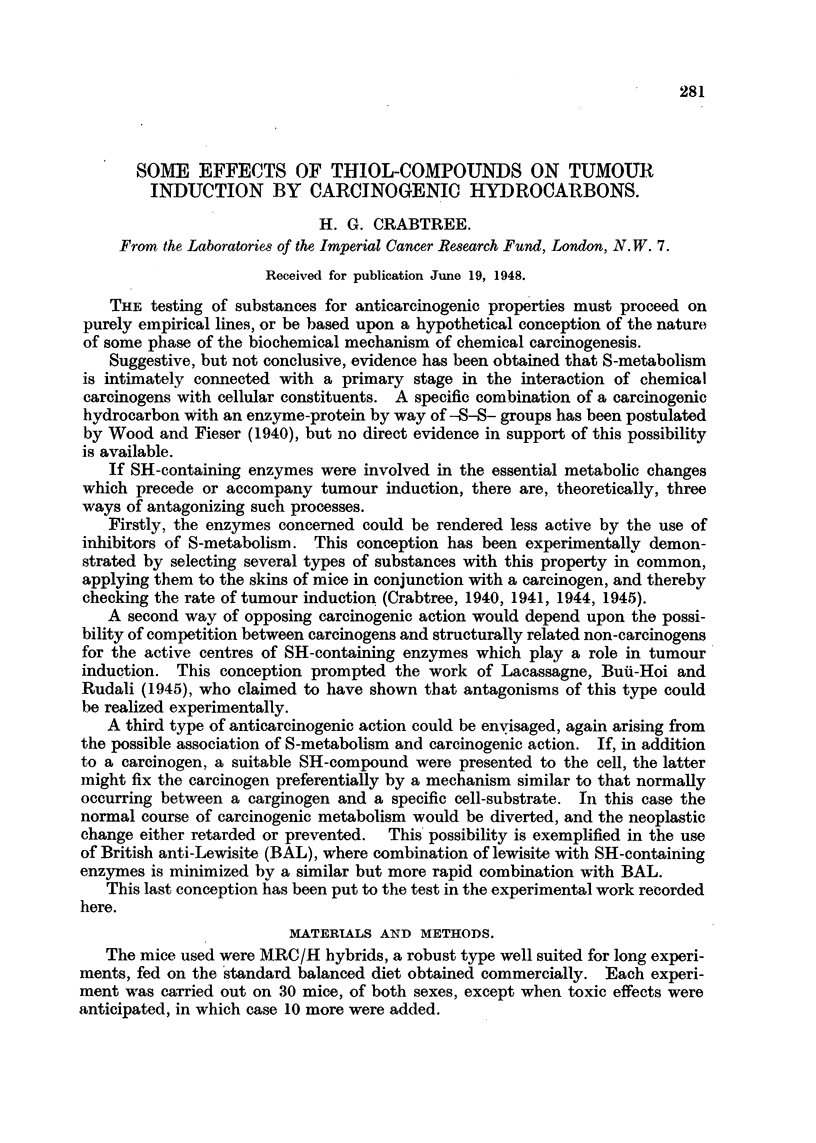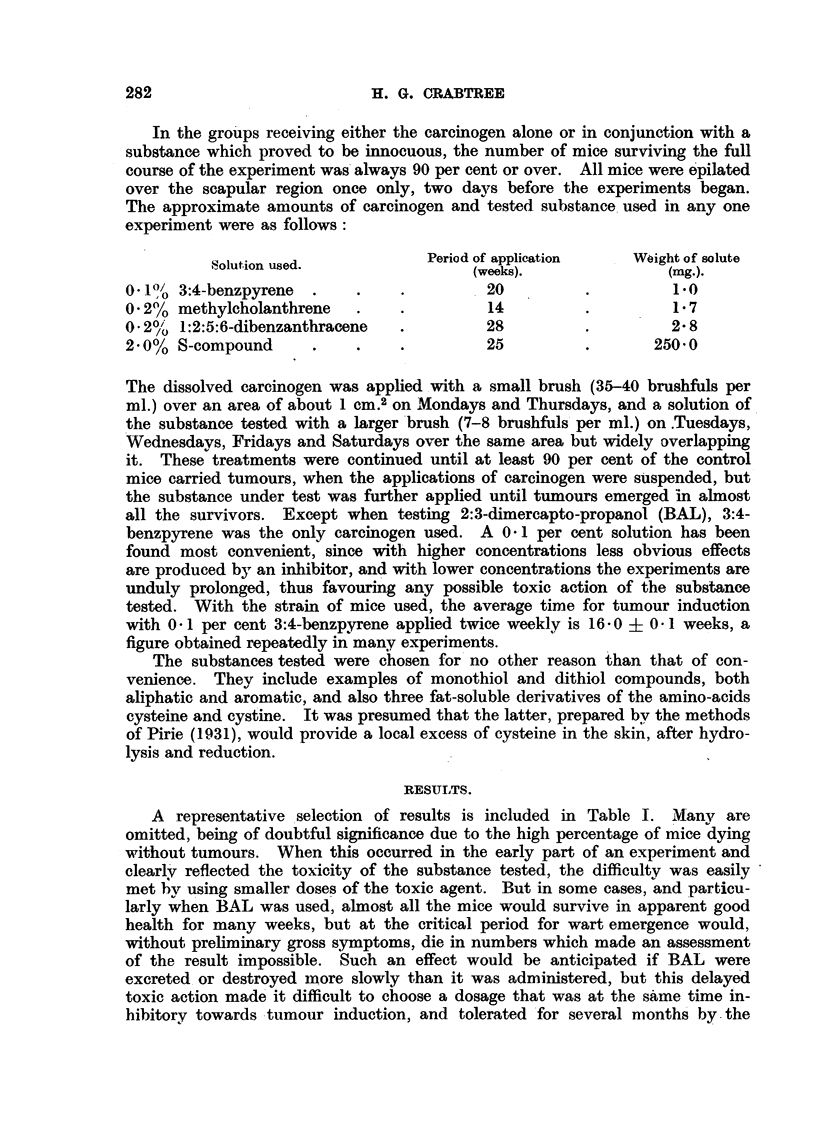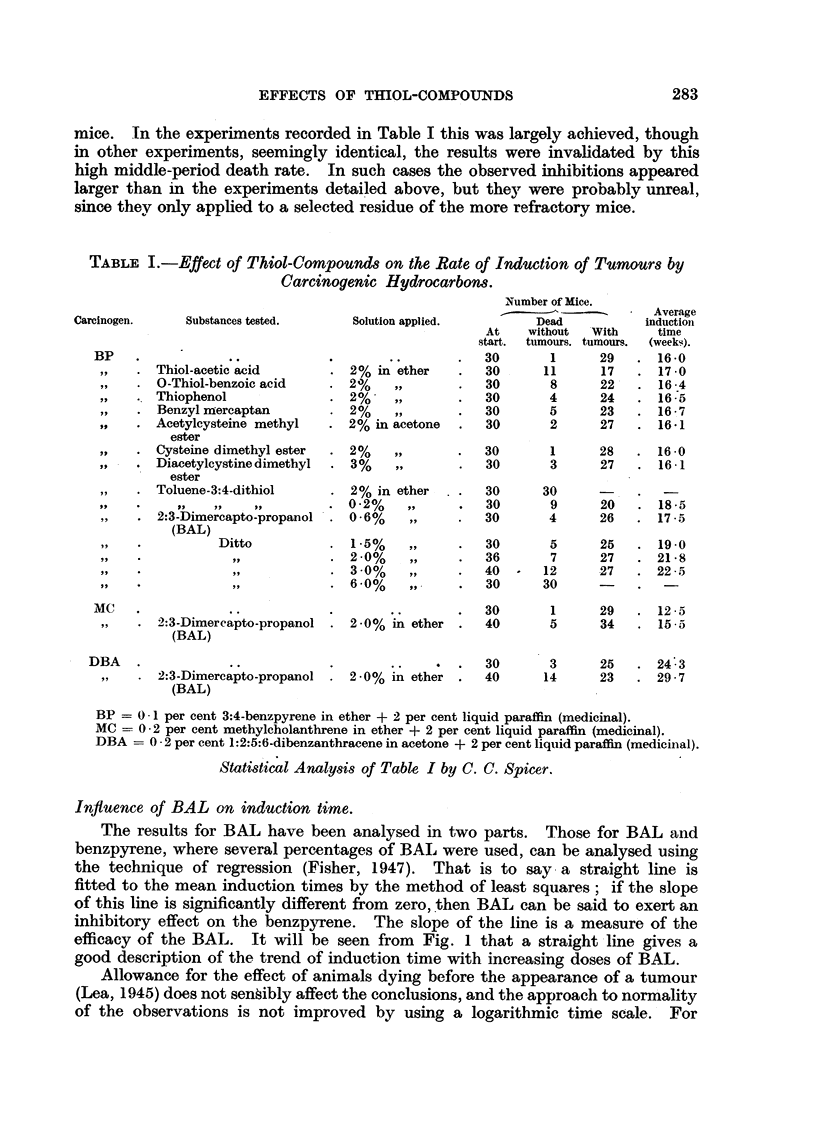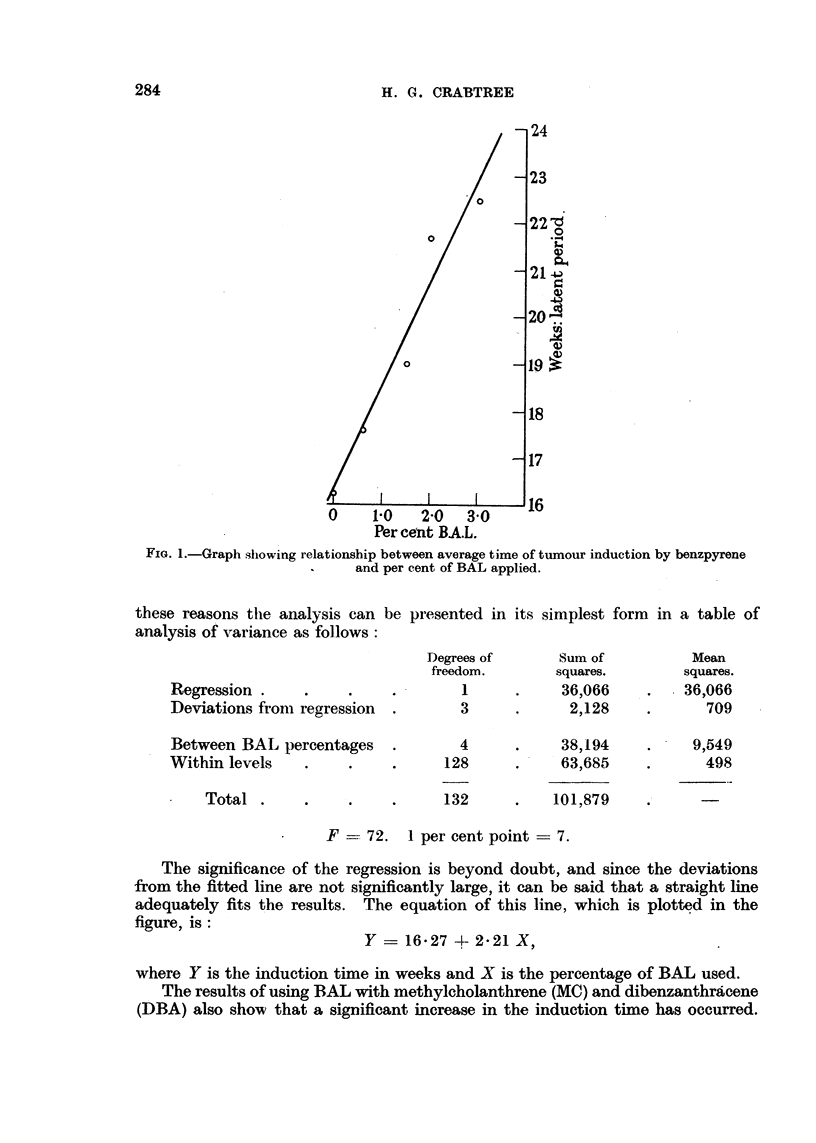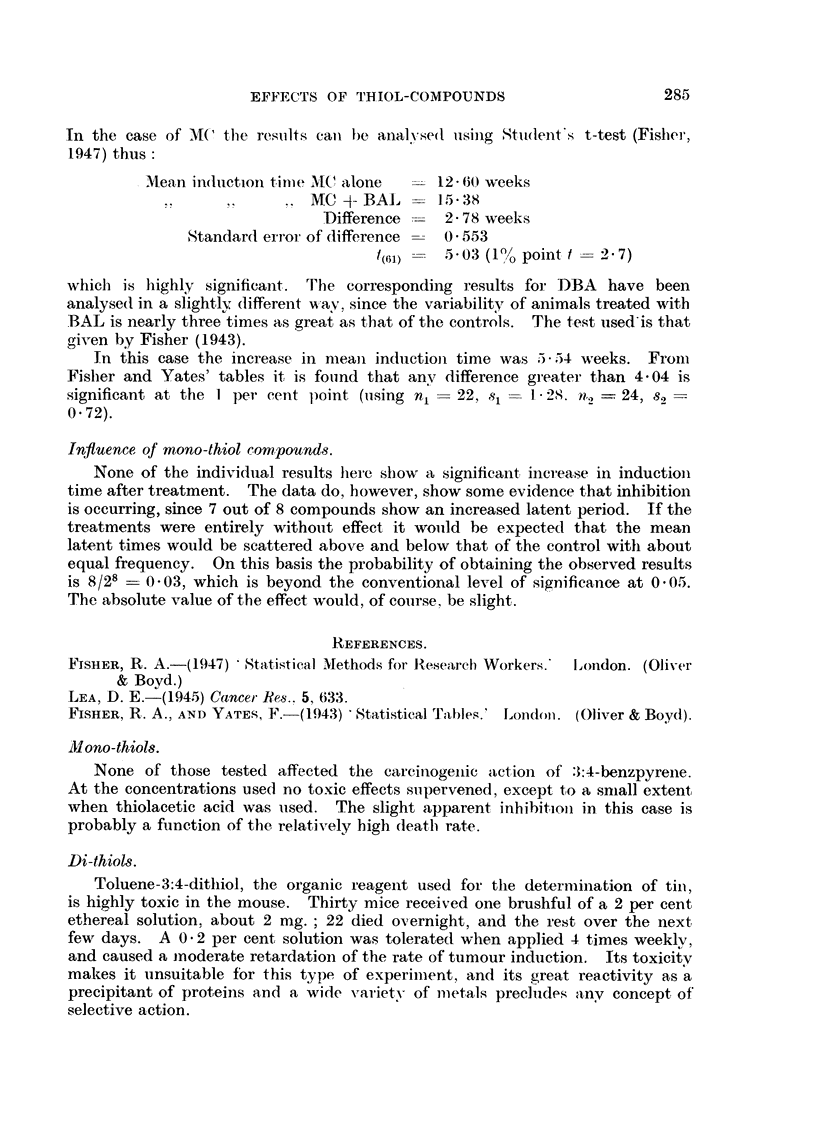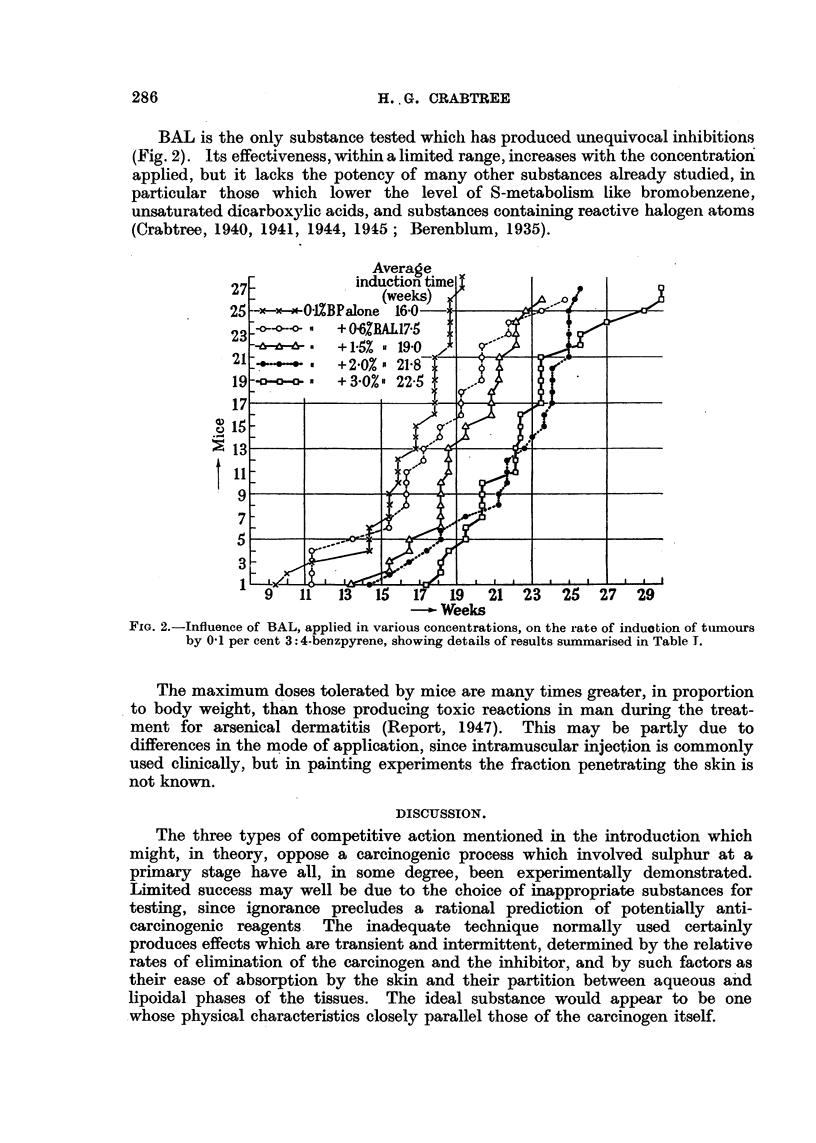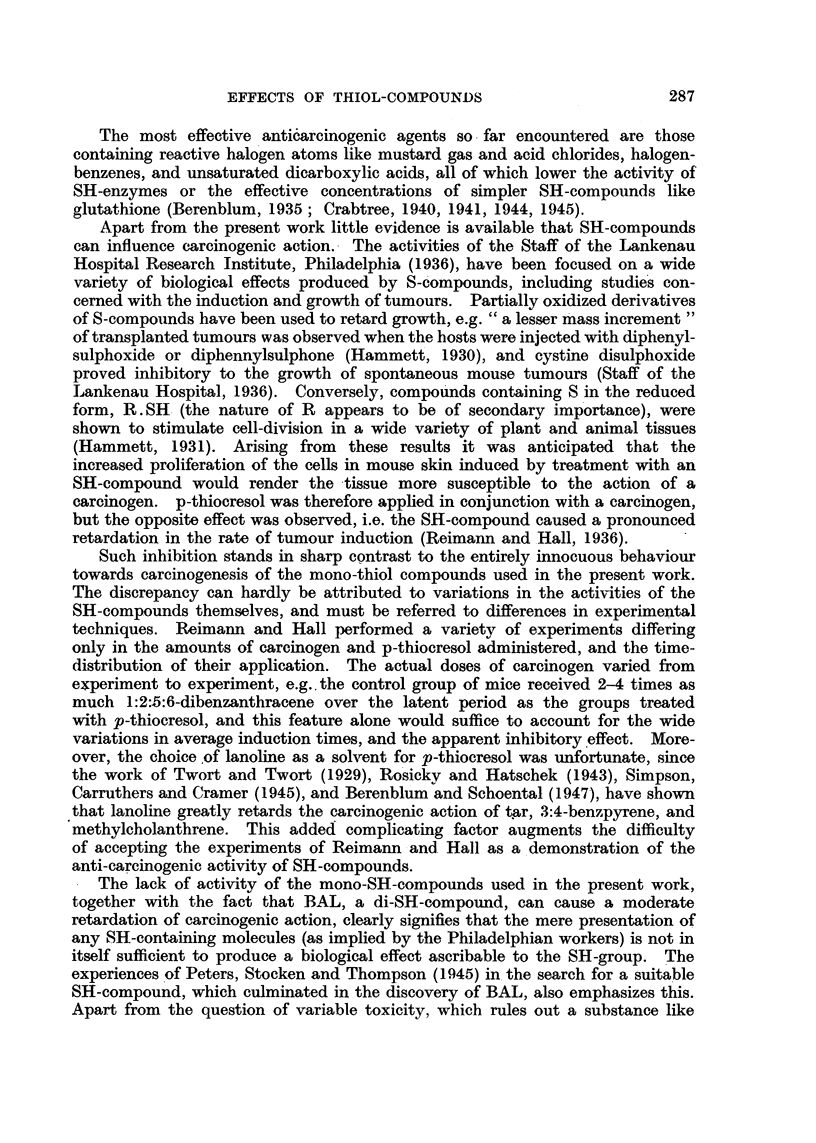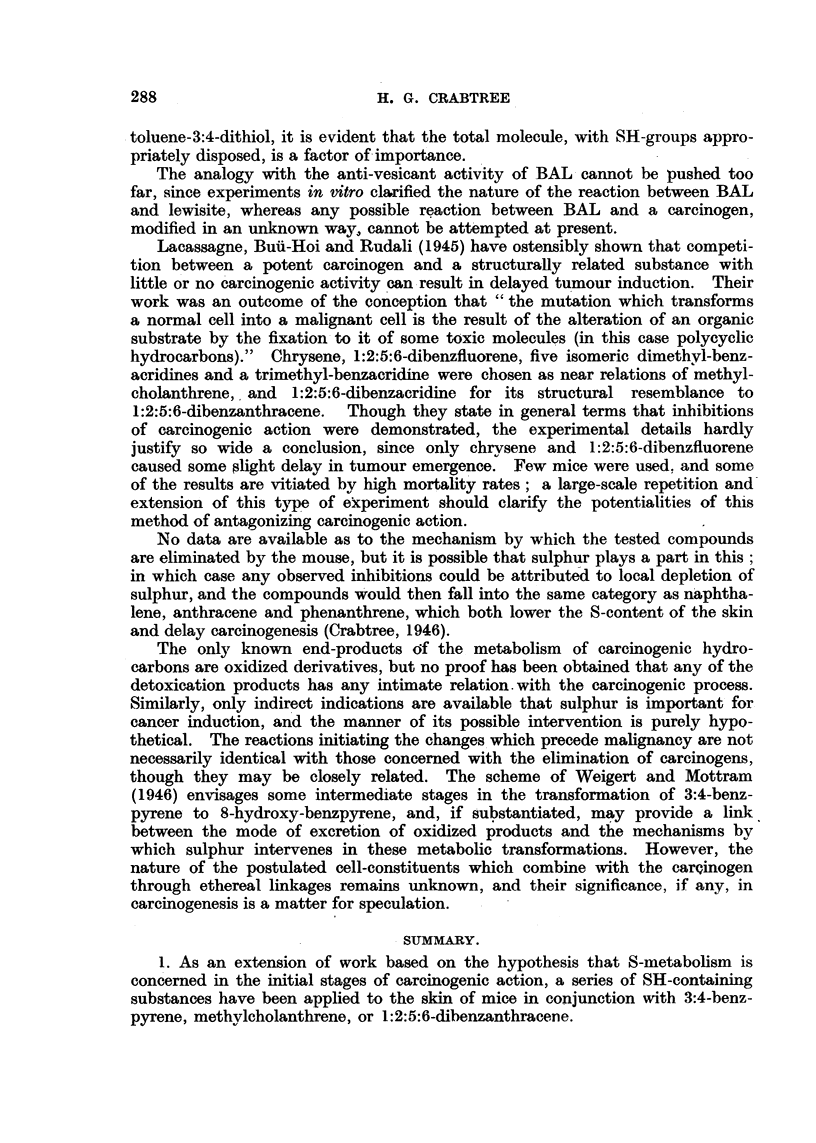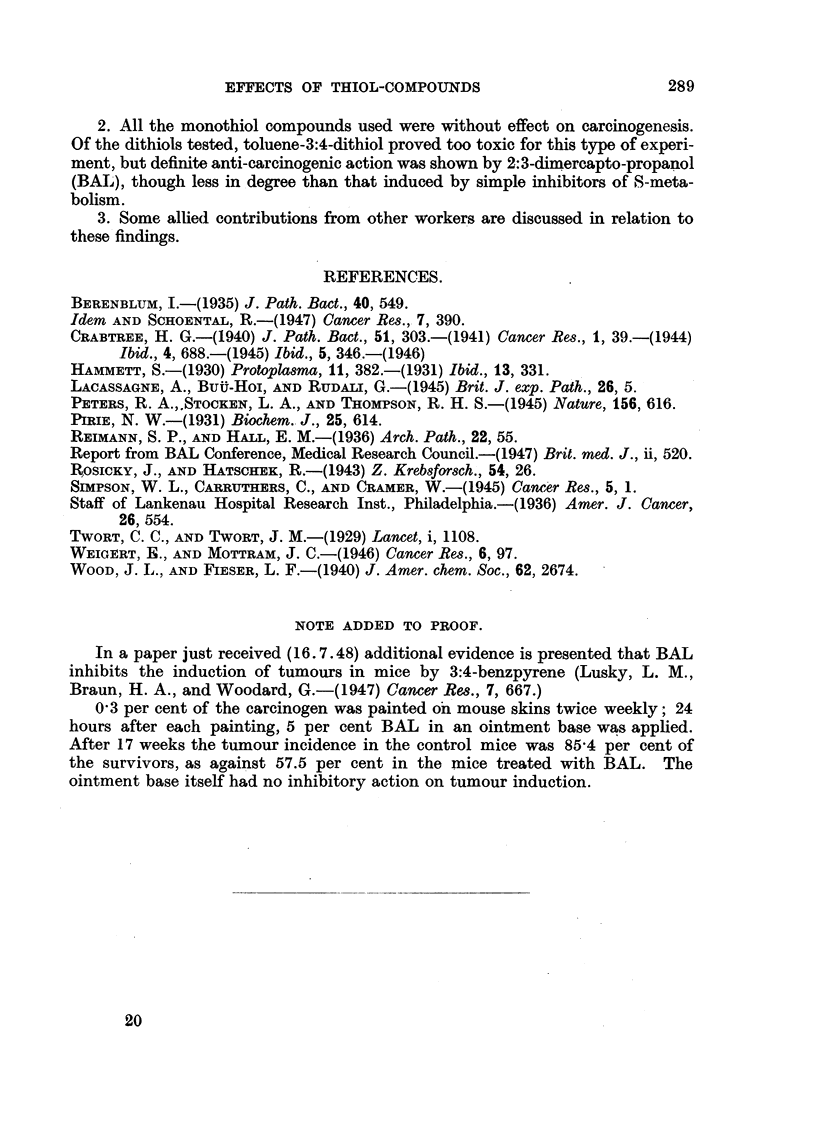# Some Effects of Thiol-Compounds on Tumour Induction by Carcinogenic Hydrocarbons

**DOI:** 10.1038/bjc.1948.33

**Published:** 1948-09

**Authors:** H. G. Crabtree


### SOME EFFECTS OF THIOL-COMPOUNDS ON TUMOUR INDUCTION BY CARCINOGENIC HYDROCARBONS.

H. G. CRABTREE.

*From the Laboratories of the Imperial Cancer Research Fund, London, N.W. 7.*

Received for publication June 19, 1948.

THE testing of substances for anticarcinogenic properties must proceed on purely empirical lines, or be based upon a hypothetical conception of the nature of some phase of the biochemical mechanism of chemical carcinogenesis.

Suggestive, but not conclusive, evidence has been obtained that S-metabolism is intimately connected with a primary stage in the interaction of chemical carcinogens with cellular constituents. A specific combination of a carcinogenic hydrocarbon with an enzyme-protein by way of –S–S– groups has been postulated by Wood and Fieser (1940), but no direct evidence in support of this possibility is available.

If SH-containing enzymes were involved in the essential metabolic changes which precede or accompany tumour induction, there are, theoretically, three ways of antagonizing such processes.

Firstly, the enzymes concerned could be rendered less active by the use of inhibitors of S-metabolism. This conception has been experimentally demonstrated by selecting several types of substances with this property in common, applying them to the skins of mice in conjunction with a carcinogen, and thereby checking the rate of tumour induction (Crabtree, 1940, 1941, 1944, 1945).

A second way of opposing carcinogenic action would depend upon the possibility of competition between carcinogens and structurally related non-carcinogens for the active centres of SH-containing enzymes which play a role in tumour induction. This conception prompted the work of Lacassagne, Buü-Hoi and Rudali (1945), who claimed to have shown that antagonisms of this type could be realized experimentally.

A third type of anticarcinogenic action could be envisaged, again arising from the possible association of S-metabolism and carcinogenic action. If, in addition to a carcinogen, a suitable SH-compound were presented to the cell, the latter might fix the carcinogen preferentially by a mechanism similar to that normally occurring between a carginogen and a specific cell-substrate. In this case the normal course of carcinogenic metabolism would be diverted, and the neoplastic change either retarded or prevented. This possibility is exemplified in the use of British anti-Lewisite (BAL), where combination of lewisite with SH-containing enzymes is minimized by a similar but more rapid combination with BAL.

This last conception has been put to the test in the experimental work recorded here.

### MATERIALS AND METHODS.

The mice used were MRC/H hybrids, a robust type well suited for long experiments, fed on the standard balanced diet obtained commercially. Each experiment was carried out on 30 mice, of both sexes, except when toxic effects were anticipated, in which case 10 more were added.

In the groups receiving either the carcinogen alone or in conjunction with a substance which proved to be innocuous, the number of mice surviving the full course of the experiment was always 90 per cent or over. All mice were epilated over the scapular region once only, two days before the experiments began. The approximate amounts of carcinogen and tested substance used in any one experiment were as follows :

| Solution used. | Period of application (weeks). | Weight of solute (mg.). |
|---|---|---|
| 0·1% 3:4-benzpyrene . . . | 20 . | 1·0 |
| 0·2% methylcholanthrene . . | 14 . | 1·7 |
| 0·2% 1:2:5:6-dibenzanthracene . | 28 . | 2·8 |
| 2·0% S-compound . . . | 25 . | 250·0 |

The dissolved carcinogen was applied with a small brush (35–40 brushfuls per ml.) over an area of about 1 cm.$^2$ on Mondays and Thursdays, and a solution of the substance tested with a larger brush (7–8 brushfuls per ml.) on Tuesdays, Wednesdays, Fridays and Saturdays over the same area but widely overlapping it. These treatments were continued until at least 90 per cent of the control mice carried tumours, when the applications of carcinogen were suspended, but the substance under test was further applied until tumours emerged in almost all the survivors. Except when testing 2:3-dimercapto-propanol (BAL), 3:4-benzpyrene was the only carcinogen used. A 0·1 per cent solution has been found most convenient, since with higher concentrations less obvious effects are produced by an inhibitor, and with lower concentrations the experiments are unduly prolonged, thus favouring any possible toxic action of the substance tested. With the strain of mice used, the average time for tumour induction with 0·1 per cent 3:4-benzpyrene applied twice weekly is $16 \cdot 0 \pm 0 \cdot 1$ weeks, a figure obtained repeatedly in many experiments.

The substances tested were chosen for no other reason than that of convenience. They include examples of monothiol and dithiol compounds, both aliphatic and aromatic, and also three fat-soluble derivatives of the amino-acids cysteine and cystine. It was presumed that the latter, prepared by the methods of Pirie (1931), would provide a local excess of cysteine in the skin, after hydrolysis and reduction.

### RESULTS.

A representative selection of results is included in Table I. Many are omitted, being of doubtful significance due to the high percentage of mice dying without tumours. When this occurred in the early part of an experiment and clearly reflected the toxicity of the substance tested, the difficulty was easily met by using smaller doses of the toxic agent. But in some cases, and particularly when BAL was used, almost all the mice would survive in apparent good health for many weeks, but at the critical period for wart emergence would, without preliminary gross symptoms, die in numbers which made an assessment of the result impossible. Such an effect would be anticipated if BAL were excreted or destroyed more slowly than it was administered, but this delayed toxic action made it difficult to choose a dosage that was at the same time inhibitory towards tumour induction, and tolerated for several months by the mice. In the experiments recorded in Table I this was largely achieved, though in other experiments, seemingly identical, the results were invalidated by this high middle-period death rate. In such cases the observed inhibitions appeared larger than in the experiments detailed above, but they were probably unreal, since they only applied to a selected residue of the more refractory mice.

TABLE I.—*Effect of Thiol-Compounds on the Rate of Induction of Tumours by Carcinogenic Hydrocarbons.*

| Carcinogen. | Substances tested. | Solution applied. | Number of Mice. At start. | Dead without tumours. | With tumours. | Average induction time (weeks). |
|---|---|---|---|---|---|---|
| BP | .. | .. | 30 | 1 | 29 | 16·0 |
| ,, | Thiol-acetic acid | 2% in ether | 30 | 11 | 17 | 17·0 |
| ,, | O-Thiol-benzoic acid | 2% ,, | 30 | 8 | 22 | 16·4 |
| ,, | Thiophenol | 2% ,, | 30 | 4 | 24 | 16·5 |
| ,, | Benzyl mercaptan | 2% ,, | 30 | 5 | 23 | 16·7 |
| ,, | Acetylcysteine methyl ester | 2% in acetone | 30 | 2 | 27 | 16·1 |
| ,, | Cysteine dimethyl ester | 2% ,, | 30 | 1 | 28 | 16·0 |
| ,, | Diacetylcystine dimethyl ester | 3% ,, | 30 | 3 | 27 | 16·1 |
| ,, | Toluene-3:4-dithiol | 2% in ether | 30 | 30 | — | — |
| ,, | ,, ,, ,, | 0·2% ,, | 30 | 9 | 20 | 18·5 |
| ,, | 2:3-Dimercapto-propanol (BAL) | 0·6% ,, | 30 | 4 | 26 | 17·5 |
| ,, | Ditto | 1·5% ,, | 30 | 5 | 25 | 19·0 |
| ,, | ,, | 2·0% ,, | 36 | 7 | 27 | 21·8 |
| ,, | ,, | 3·0% ,, | 40 | 12 | 27 | 22·5 |
| ,, | ,, | 6·0% ,, | 30 | 30 | — | — |
| MC | .. | .. | 30 | 1 | 29 | 12·5 |
| ,, | 2:3-Dimercapto-propanol (BAL) | 2·0% in ether | 40 | 5 | 34 | 15·5 |
| DBA | .. | .. | 30 | 3 | 25 | 24·3 |
| ,, | 2:3-Dimercapto-propanol (BAL) | 2·0% in ether | 40 | 14 | 23 | 29·7 |

BP = 0·1 per cent 3:4-benzpyrene in ether + 2 per cent liquid paraffin (medicinal).
MC = 0·2 per cent methylcholanthrene in ether + 2 per cent liquid paraffin (medicinal).
DBA = 0·2 per cent 1:2:5:6-dibenzanthracene in acetone + 2 per cent liquid paraffin (medicinal).

*Statistical Analysis of Table I by C. C. Spicer.*

*Influence of BAL on induction time.*

The results for BAL have been analysed in two parts. Those for BAL and benzpyrene, where several percentages of BAL were used, can be analysed using the technique of regression (Fisher, 1947). That is to say a straight line is fitted to the mean induction times by the method of least squares ; if the slope of this line is significantly different from zero, then BAL can be said to exert an inhibitory effect on the benzpyrene. The slope of the line is a measure of the efficacy of the BAL. It will be seen from Fig. 1 that a straight line gives a good description of the trend of induction time with increasing doses of BAL.

Allowance for the effect of animals dying before the appearance of a tumour (Lea, 1945) does not sensibly affect the conclusions, and the approach to normality of the observations is not improved by using a logarithmic time scale. For

FIG. 1.—Graph showing relationship between average time of tumour induction by benzpyrene and per cent of BAL applied.

these reasons the analysis can be presented in its simplest form in a table of analysis of variance as follows :

| | Degrees of freedom. | Sum of squares. | Mean squares. |
|---|---|---|---|
| Regression . . . . . | 1 | 36,066 | 36,066 |
| Deviations from regression . | 3 | 2,128 | 709 |
| Between BAL percentages . | 4 | 38,194 | 9,549 |
| Within levels . . . | 128 | 63,685 | 498 |
| Total . . . . | 132 | 101,879 | — |

$F = 72$. 1 per cent point $= 7$.

The significance of the regression is beyond doubt, and since the deviations from the fitted line are not significantly large, it can be said that a straight line adequately fits the results. The equation of this line, which is plotted in the figure, is :

$$Y = 16 \cdot 27 + 2 \cdot 21\ X,$$

where $Y$ is the induction time in weeks and $X$ is the percentage of BAL used.

The results of using BAL with methylcholanthrene (MC) and dibenzanthrăcene (DBA) also show that a significant increase in the induction time has occurred.

In the case of MC the results can be analysed using Student's t-test (Fisher, 1947) thus :

| | | |
|---|---|---|
| Mean induction time MC alone | = | 12·60 weeks |
| ,, ,, ,, MC + BAL | = | 15·38 |
| Difference | = | 2·78 weeks |
| Standard error of difference | = | 0·553 |
| $t_{(61)}$ | = | 5·03 (1% point $t$ = 2·7) |

which is highly significant. The corresponding results for DBA have been analysed in a slightly different way, since the variability of animals treated with BAL is nearly three times as great as that of the controls. The test used is that given by Fisher (1943).

In this case the increase in mean induction time was 5·54 weeks. From Fisher and Yates' tables it is found that any difference greater than 4·04 is significant at the 1 per cent point (using $n_1 = 22$, $s_1 = 1·28$, $n_2 = 24$, $s_2 = 0·72$).

*Influence of mono-thiol compounds.*

None of the individual results here show a significant increase in induction time after treatment. The data do, however, show some evidence that inhibition is occurring, since 7 out of 8 compounds show an increased latent period. If the treatments were entirely without effect it would be expected that the mean latent times would be scattered above and below that of the control with about equal frequency. On this basis the probability of obtaining the observed results is $8/2^8 = 0·03$, which is beyond the conventional level of significance at 0·05. The absolute value of the effect would, of course, be slight.

### References.

FISHER, R. A.—(1947) 'Statistical Methods for Research Workers.' London. (Oliver & Boyd.)
LEA, D. E.—(1945) *Cancer Res.*, **5**, 633.
FISHER, R. A., AND YATES, F.—(1943) 'Statistical Tables.' London. (Oliver & Boyd).

*Mono-thiols.*

None of those tested affected the carcinogenic action of 3:4-benzpyrene. At the concentrations used no toxic effects supervened, except to a small extent when thiolacetic acid was used. The slight apparent inhibition in this case is probably a function of the relatively high death rate.

*Di-thiols.*

Toluene-3:4-dithiol, the organic reagent used for the determination of tin, is highly toxic in the mouse. Thirty mice received one brushful of a 2 per cent ethereal solution, about 2 mg. ; 22 died overnight, and the rest over the next few days. A 0·2 per cent solution was tolerated when applied 4 times weekly, and caused a moderate retardation of the rate of tumour induction. Its toxicity makes it unsuitable for this type of experiment, and its great reactivity as a precipitant of proteins and a wide variety of metals precludes any concept of selective action.

BAL is the only substance tested which has produced unequivocal inhibitions (Fig. 2). Its effectiveness, within a limited range, increases with the concentration applied, but it lacks the potency of many other substances already studied, in particular those which lower the level of S-metabolism like bromobenzene, unsaturated dicarboxylic acids, and substances containing reactive halogen atoms (Crabtree, 1940, 1941, 1944, 1945; Berenblum, 1935).

FIG. 2.—Influence of BAL, applied in various concentrations, on the rate of induction of tumours by 0·1 per cent 3:4-benzpyrene, showing details of results summarised in Table I.

The maximum doses tolerated by mice are many times greater, in proportion to body weight, than those producing toxic reactions in man during the treatment for arsenical dermatitis (Report, 1947). This may be partly due to differences in the mode of application, since intramuscular injection is commonly used clinically, but in painting experiments the fraction penetrating the skin is not known.

### DISCUSSION.

The three types of competitive action mentioned in the introduction which might, in theory, oppose a carcinogenic process which involved sulphur at a primary stage have all, in some degree, been experimentally demonstrated. Limited success may well be due to the choice of inappropriate substances for testing, since ignorance precludes a rational prediction of potentially anti-carcinogenic reagents The inadequate technique normally used certainly produces effects which are transient and intermittent, determined by the relative rates of elimination of the carcinogen and the inhibitor, and by such factors as their ease of absorption by the skin and their partition between aqueous and lipoidal phases of the tissues. The ideal substance would appear to be one whose physical characteristics closely parallel those of the carcinogen itself.

The most effective anticarcinogenic agents so far encountered are those containing reactive halogen atoms like mustard gas and acid chlorides, halogen-benzenes, and unsaturated dicarboxylic acids, all of which lower the activity of SH-enzymes or the effective concentrations of simpler SH-compounds like glutathione (Berenblum, 1935 ; Crabtree, 1940, 1941, 1944, 1945).

Apart from the present work little evidence is available that SH-compounds can influence carcinogenic action. The activities of the Staff of the Lankenau Hospital Research Institute, Philadelphia (1936), have been focused on a wide variety of biological effects produced by S-compounds, including studies concerned with the induction and growth of tumours. Partially oxidized derivatives of S-compounds have been used to retard growth, e.g. " a lesser mass increment " of transplanted tumours was observed when the hosts were injected with diphenylsulphoxide or diphennylsulphone (Hammett, 1930), and cystine disulphoxide proved inhibitory to the growth of spontaneous mouse tumours (Staff of the Lankenau Hospital, 1936). Conversely, compounds containing S in the reduced form, R.SH (the nature of R appears to be of secondary importance), were shown to stimulate cell-division in a wide variety of plant and animal tissues (Hammett, 1931). Arising from these results it was anticipated that the increased proliferation of the cells in mouse skin induced by treatment with an SH-compound would render the tissue more susceptible to the action of a carcinogen. p-thiocresol was therefore applied in conjunction with a carcinogen, but the opposite effect was observed, i.e. the SH-compound caused a pronounced retardation in the rate of tumour induction (Reimann and Hall, 1936).

Such inhibition stands in sharp contrast to the entirely innocuous behaviour towards carcinogenesis of the mono-thiol compounds used in the present work. The discrepancy can hardly be attributed to variations in the activities of the SH-compounds themselves, and must be referred to differences in experimental techniques. Reimann and Hall performed a variety of experiments differing only in the amounts of carcinogen and p-thiocresol administered, and the time-distribution of their application. The actual doses of carcinogen varied from experiment to experiment, e.g. the control group of mice received 2–4 times as much 1:2:5:6-dibenzanthracene over the latent period as the groups treated with *p*-thiocresol, and this feature alone would suffice to account for the wide variations in average induction times, and the apparent inhibitory effect. Moreover, the choice of lanoline as a solvent for *p*-thiocresol was unfortunate, since the work of Twort and Twort (1929), Rosicky and Hatschek (1943), Simpson, Carruthers and Cramer (1945), and Berenblum and Schoental (1947), have shown that lanoline greatly retards the carcinogenic action of tar, 3:4-benzpyrene, and methylcholanthrene. This added complicating factor augments the difficulty of accepting the experiments of Reimann and Hall as a demonstration of the anti-carcinogenic activity of SH-compounds.

The lack of activity of the mono-SH-compounds used in the present work, together with the fact that BAL, a di-SH-compound, can cause a moderate retardation of carcinogenic action, clearly signifies that the mere presentation of any SH-containing molecules (as implied by the Philadelphian workers) is not in itself sufficient to produce a biological effect ascribable to the SH-group. The experiences of Peters, Stocken and Thompson (1945) in the search for a suitable SH-compound, which culminated in the discovery of BAL, also emphasizes this. Apart from the question of variable toxicity, which rules out a substance like toluene-3:4-dithiol, it is evident that the total molecule, with SH-groups appropriately disposed, is a factor of importance.

The analogy with the anti-vesicant activity of BAL cannot be pushed too far, since experiments *in vitro* clarified the nature of the reaction between BAL and lewisite, whereas any possible reaction between BAL and a carcinogen, modified in an unknown way, cannot be attempted at present.

Lacassagne, Buü-Hoi and Rudali (1945) have ostensibly shown that competition between a potent carcinogen and a structurally related substance with little or no carcinogenic activity can result in delayed tumour induction. Their work was an outcome of the conception that " the mutation which transforms a normal cell into a malignant cell is the result of the alteration of an organic substrate by the fixation to it of some toxic molecules (in this case polycyclic hydrocarbons)." Chrysene, 1:2:5:6-dibenzfluorene, five isomeric dimethyl-benzacridines and a trimethyl-benzacridine were chosen as near relations of methylcholanthrene, and 1:2:5:6-dibenzacridine for its structural resemblance to 1:2:5:6-dibenzanthracene. Though they state in general terms that inhibitions of carcinogenic action were demonstrated, the experimental details hardly justify so wide a conclusion, since only chrysene and 1:2:5:6-dibenzfluorene caused some slight delay in tumour emergence. Few mice were used, and some of the results are vitiated by high mortality rates ; a large-scale repetition and extension of this type of experiment should clarify the potentialities of this method of antagonizing carcinogenic action.

No data are available as to the mechanism by which the tested compounds are eliminated by the mouse, but it is possible that sulphur plays a part in this ; in which case any observed inhibitions could be attributed to local depletion of sulphur, and the compounds would then fall into the same category as naphthalene, anthracene and phenanthrene, which both lower the S-content of the skin and delay carcinogenesis (Crabtree, 1946).

The only known end-products of the metabolism of carcinogenic hydrocarbons are oxidized derivatives, but no proof has been obtained that any of the detoxication products has any intimate relation with the carcinogenic process. Similarly, only indirect indications are available that sulphur is important for cancer induction, and the manner of its possible intervention is purely hypothetical. The reactions initiating the changes which precede malignancy are not necessarily identical with those concerned with the elimination of carcinogens, though they may be closely related. The scheme of Weigert and Mottram (1946) envisages some intermediate stages in the transformation of 3:4-benzpyrene to 8-hydroxy-benzpyrene, and, if substantiated, may provide a link between the mode of excretion of oxidized products and the mechanisms by which sulphur intervenes in these metabolic transformations. However, the nature of the postulated cell-constituents which combine with the carcinogen through ethereal linkages remains unknown, and their significance, if any, in carcinogenesis is a matter for speculation.

### SUMMARY.

1. As an extension of work based on the hypothesis that S-metabolism is concerned in the initial stages of carcinogenic action, a series of SH-containing substances have been applied to the skin of mice in conjunction with 3:4-benzpyrene, methylcholanthrene, or 1:2:5:6-dibenzanthracene.

2. All the monothiol compounds used were without effect on carcinogenesis. Of the dithiols tested, toluene-3:4-dithiol proved too toxic for this type of experiment, but definite anti-carcinogenic action was shown by 2:3-dimercapto-propanol (BAL), though less in degree than that induced by simple inhibitors of S-metabolism.

3. Some allied contributions from other workers are discussed in relation to these findings.

### REFERENCES.

BERENBLUM, I.—(1935) *J. Path. Bact.*, **40**, 549.
*Idem* AND SCHOENTAL, R.—(1947) *Cancer Res.*, **7**, 390.
CRABTREE, H. G.—(1940) *J. Path. Bact.*, **51**, 303.—(1941) *Cancer Res.*, **1**, 39.—(1944) *Ibid.*, **4**, 688.—(1945) *Ibid.*, **5**, 346.—(1946)
HAMMETT, S.—(1930) *Protoplasma*, **11**, 382.—(1931) *Ibid.*, **13**, 331.
LACASSAGNE, A., BUÜ-HOI, AND RUDALI, G.—(1945) *Brit. J. exp. Path.*, **26**, 5.
PETERS, R. A., STOCKEN, L. A., AND THOMPSON, R. H. S.—(1945) *Nature*, **156**, 616.
PIRIE, N. W.—(1931) *Biochem. J.*, **25**, 614.
REIMANN, S. P., AND HALL, E. M.—(1936) *Arch. Path.*, **22**, 55.
Report from BAL Conference, Medical Research Council.—(1947) *Brit. med. J.*, ii, 520.
ROSICKY, J., AND HATSCHEK, R.—(1943) *Z. Krebsforsch.*, **54**, 26.
SIMPSON, W. L., CARRUTHERS, C., AND CRAMER, W.—(1945) *Cancer Res.*, **5**, 1.
Staff of Lankenau Hospital Research Inst., Philadelphia.—(1936) *Amer. J. Cancer*, **26**, 554.
TWORT, C. C., AND TWORT, J. M.—(1929) *Lancet*, i, 1108.
WEIGERT, E., AND MOTTRAM, J. C.—(1946) *Cancer Res.*, **6**, 97.
WOOD, J. L., AND FIESER, L. F.—(1940) *J. Amer. chem. Soc.*, **62**, 2674.

### NOTE ADDED TO PROOF.

In a paper just received (16.7.48) additional evidence is presented that BAL inhibits the induction of tumours in mice by 3:4-benzpyrene (Lusky, L. M., Braun, H. A., and Woodard, G.—(1947) *Cancer Res.*, 7, 667.)

0·3 per cent of the carcinogen was painted on mouse skins twice weekly; 24 hours after each painting, 5 per cent BAL in an ointment base was applied. After 17 weeks the tumour incidence in the control mice was 85·4 per cent of the survivors, as against 57.5 per cent in the mice treated with BAL. The ointment base itself had no inhibitory action on tumour induction.